# Dosimetry with gafchromic films based on a new micro-opto-electro-mechanical system

**DOI:** 10.1038/s41598-021-89602-9

**Published:** 2021-05-17

**Authors:** C. Guardiola, A. Márquez, M. C. Jiménez-Ramos, J. García López, A. Baratto-Roldán, X. Muñoz-Berbel

**Affiliations:** 1 Université Paris‒Saclay, CNRS/IN2P3, IJCLab, 91405 Orsay, France; 2 Instituto de Microelectrónica de Barcelona, (IMB-CNM, CSIC), 08193 Bellaterra, Spain; 3 Centro Nacional de Aceleradores, 41092 Sevilla, Spain; 4 Department of Atomic, Molecular and Nuclear Physics, Universidad de Sevilla, 41012 Sevilla, Spain

**Keywords:** Radiotherapy, Applied physics

## Abstract

This work presents the first tests performed with radiochromic films and a new Micro‒Opto‒Electro-Mechanical system (MOEMS) for in situ dosimetry evaluation in radiotherapy in real time. We present a new device and methodology that overcomes the traditional limitation of time-delay in radiochromic film analysis by turning a passive detector into an active sensor. The proposed system consists mainly of an optical sensor based on light emitting diodes and photodetectors controlled by both customized electronic circuit and graphical user interface, which enables optical measurements directly. We show the first trials performed in a low‒energy proton cyclotron with this MOEMS by using gafchromic EBT3 films. Results show the feasibility of using this system for in situ dose evaluations. Further adaptation is ongoing to develop a full real‒time active detector by integrating MOEM multi‒arrays and films in flexible printed circuits. Hence, we point to improve the clinical application of radiochromic films with the aim to optimize radiotherapy treatment verifications.

## Introduction

In radiotherapy, a precise evaluation of the dose delivered to the patients is fundamental. Radiochromic films are solid state detectors that have been traditionally considered the basic 2D passive detectors in the area for dose verification from tens of mGy to hundreds of Gy for diagnostic instrumentation, e.g. X‒ray radiography, computer tomography, mammography, i.a., and determination of depth‒dose profiles in photon, electron and ion beams. Due to their high spatial resolution, low energy dependence, and quasi water‒equivalence, they are widely used for quality assurance (QA) measurements and treatment verification^1–6^. There are several kind of films that are employed in photon dosimetry^7–9^ due to their energy and dose‒rate independence and nearly tissue equivalence ^10^. However, in hadron irradiations, they have an energy dependence in the Bragg peak^11,12^, showing an under‒response in dose in the high lineal energy transfer regions, which requires the use of correction factors of that quenching effect^13^. Even so, they are indeed the gold‒standard passive detector in radiation therapy^14–16^. Under exposure to ionizing radiation, a polymerization reaction occurs within the active layer of the radiochromic film and it turns blue (chromatic change) proportionally to the radiation dose^17^. This chemical reaction produces an irreversible change in the optical density (OD) of the film and may be correlated to the delivered dose inside it without additional chemical post‒processing. The netOD of a film represents the difference of OD after and before of being irradiated. However, the standard quantification method of those OD changes, which is based on image scanning by using densitometers, e.g. flatbed scanner, requires a post‒processing after irradiation. Indeed, a minimum time of 24 h is recommended between irradiation moment and film scanning^1,10,18^. Therefore, there is a considerable time‒delay for evaluating the dose delivered. This analysis time‒delay makes radiochromic films an unfeasible tool for real‒time and in situ daily dose evaluations provided OD is considered as the only parameter to be correlated to the delivered dose. In practice, when it is mandatory to provide a dose verification in real time, the films are complemented with active radiation sensors as semiconductor or scintillation detectors, gas chambers, i.a.^19–22^.

In response to these issues, we aimed at creating a novel device by using another physical parameter as well as a new method to obtain the dose derived from an innovative Micro-Opto-Electro-Mechanical system (MOEMS)^23–27^. The system resembles those already produced by our group^28,29^. It consists of an optical sensor with light emitting diodes and photodetectors integrated in a mechanical and electrical system. A patent has been filled in March 2021^30^. We present the first experimental tests performed in a proton cyclotron with this MOEMS by using gafchromic EBT3 films, which belong to the film model widely used in particle therapy for external beam therapy^31^. Results show the feasibility of using this system for further real‒time dose evaluation.

In this context, there are a few proposals based on fiber optic probes in real‒time, but they require large and bulky instruments (light sources and spectrometers) and are limited in spatial resolution, which is not practical for daily dose verifications^32–35^. However, our approach consists in a portable electro-mechanical setup whose configuration allows us to reply the sensitive area to cover several centimeters. It also includes a graphical user interface for direct data display, as detailed below. To the best of our knowledge, this is the first MOEMS to overcome the traditional limitation of time‒delay in gafchromic film analysis by turning a passive detector into a potential active sensing system in radiotherapy.

## Methods

Dose evaluations were characterized with gafchromic films^1^, which are crystalline polyacetylene based radiochromic films^2,9,15^. In this work, we use films of the EBT model that was designed for external beam therapy, radiosurgery and brachytherapy QA (GAFCHROMIC, International Specialty Products, Wayne, New Jersey, USA)^31^. It contains a yellow marker dye inside the sensitive layer and shows a high sensitivity compared to previous models^16^. The optimal dose range of these films is between 0.2 Gy and 10 Gy and they have uniformities better than ± 3% in dose. Additionally, they are coated on special polyester to avoid Newton’s rings patterns and show a spatial resolution up to 25 µm^31^.

### Micro-opto-electro-mechanical system

**Figure 1:**
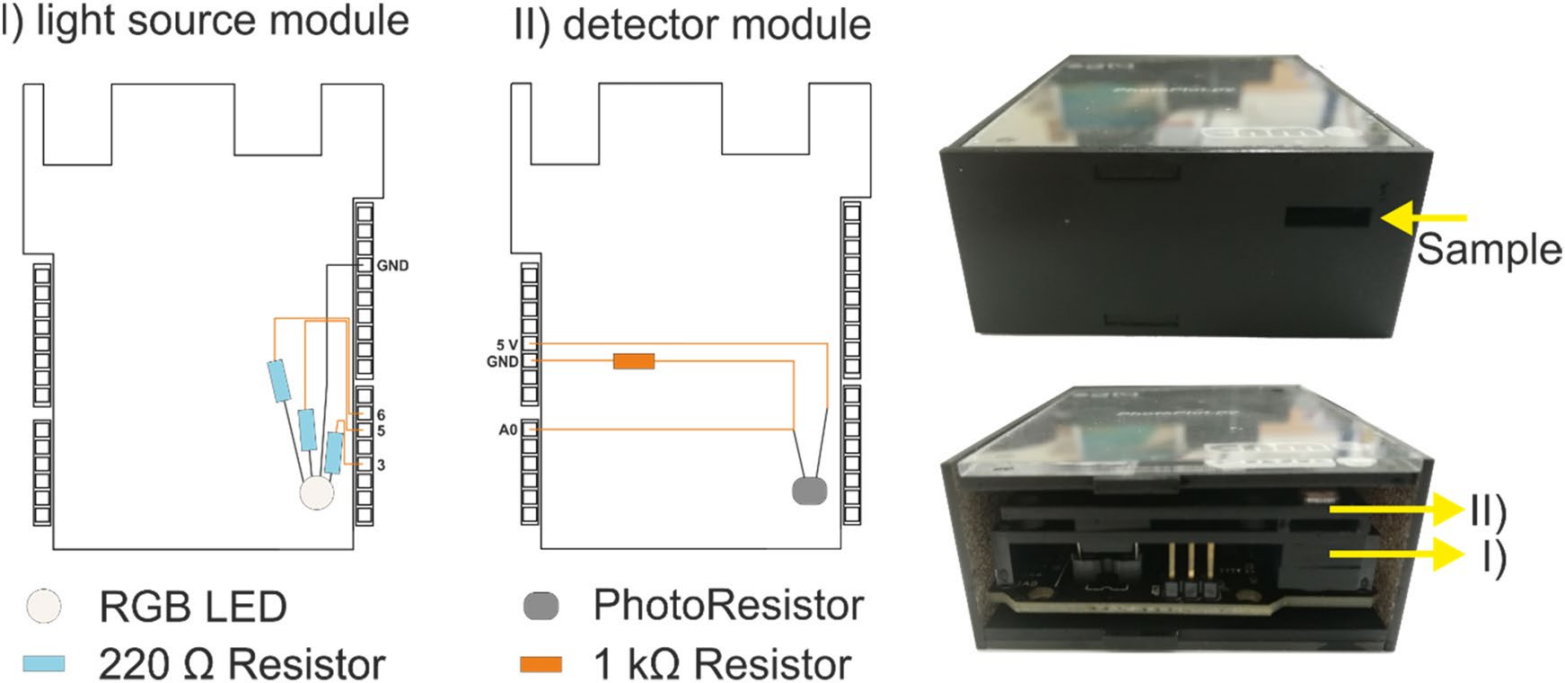
Sketch of the two modules of the optical sensing system. Left: LED light-emitting module. Center: Detector module consisting mainly of a photoresistor and 1 kΩ resistor. Right: Image of both assembled modules. *Elegoo Uno* boards are not shown in the sketch.

The sensing system used is a MOEMS developed by the Chemical Transducers group in the National Center of Microelectronics at Barcelona (IMB‒CNM, CSIC). It consists of an *Elegoo Uno* board and two dedicated modules, namely (i) one module with light emitting diodes, i.e. LEDs (RGB), that may be activated independently, and (ii) another module with a light-dependent resistor (LDR). The former is the light source and the second one works as a photodetector. Figure 1 shows a simplified sketch of it.

**Figure 2:**
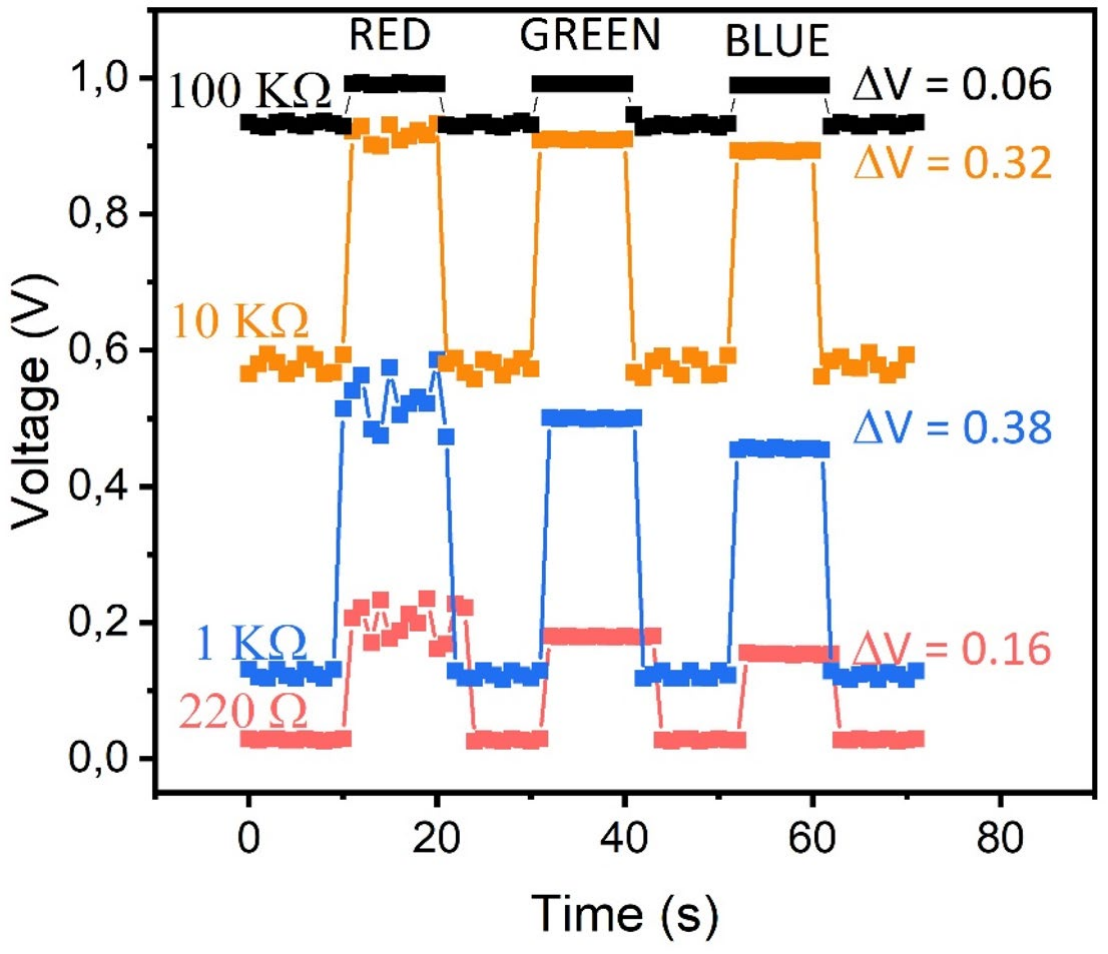
Voltage measured (V) versus time (s) for a set of resistances with four orders of magnitudes of difference (0.22, 1, 10 and 100 kΩ). The highest voltage gradient is displayed for the 1kΩ resistance value.

The module platforms, as well as the device black cage, are made of poly(methyl methacrylate) (PMMA) sheets, cut by CO_2_ laser ablation system. The electronic compounds (tin welded) are adjusted to the modules cavities and fixed with non‒conductive epoxy. In the light transmitter module, every LED of the RGB set is connected to the board pins limiting the current with a 220 Ω resistance for a better control of the light intensity, which can be adjusted using the developed software interface. Samples are put in a mechanic cavity (1×8×20 cm) between both modules individually. When the LED (RGB) module is activated, i.e. one or a combination of various RBG emitters, the corresponding RGB light goes through the sample. The transmitted light reaches then the detector module and provokes a resistance decay of the LDR, which is inversely proportional to the amount of light received. Specifically, the used LDR offers a resistance of 500 kΩ in darkness and only 500 Ω in light‒saturation conditions. In order to read the on‒the‒spot resistance, the signal must be converted to a potential value. The chosen way to do that was using a fixed resistor, as it is depicted in Fig. 1. The 5 V source, photoresistor, 1 kΩ resistor and ground are connected in series, in this order. Between the photoresistor and the 1 kΩ resistor, an analog voltage reading (A0) is connected. In such configuration, as the resistance of the photocell decreases, the current through both resistors increases, what causes a voltage rise across the 1 kΩ fixed resistor (following the Ohm’s law V = R·I). The chosen value of the fixed resistor was optimized in order to obtain the highest sensitivity with the measured samples. We performed a study testing different resistors to obtain the one giving the best response (in ∆V) in our system. Figure 2 shows the voltage variations when it is turned on and off each of the RGB LEDs for a set of resistances covering four orders of magnitudes (0.22, 1, 10 and 100 kΩ).

The 1 kΩ resistance generates the highest gradient in voltage. Therefore, it would yield the better sensitivity regarding changes in voltage that are directly related to the resolution of our system.

The communication system is programming with *Python* (an open‒source electronics platform) and managed with in‒house python‒based software, using a graphic user interface developed with the Tkinter package.

Following that method, measurements with red, green, and both lights were performed systematically. We repeated these measurements 10 min, 1, 6, 24 and 48 hours after irradiation to study the possible voltage changes along the time. Results are shown below.

### Spectrophotometer analysis

**Figure 3:**
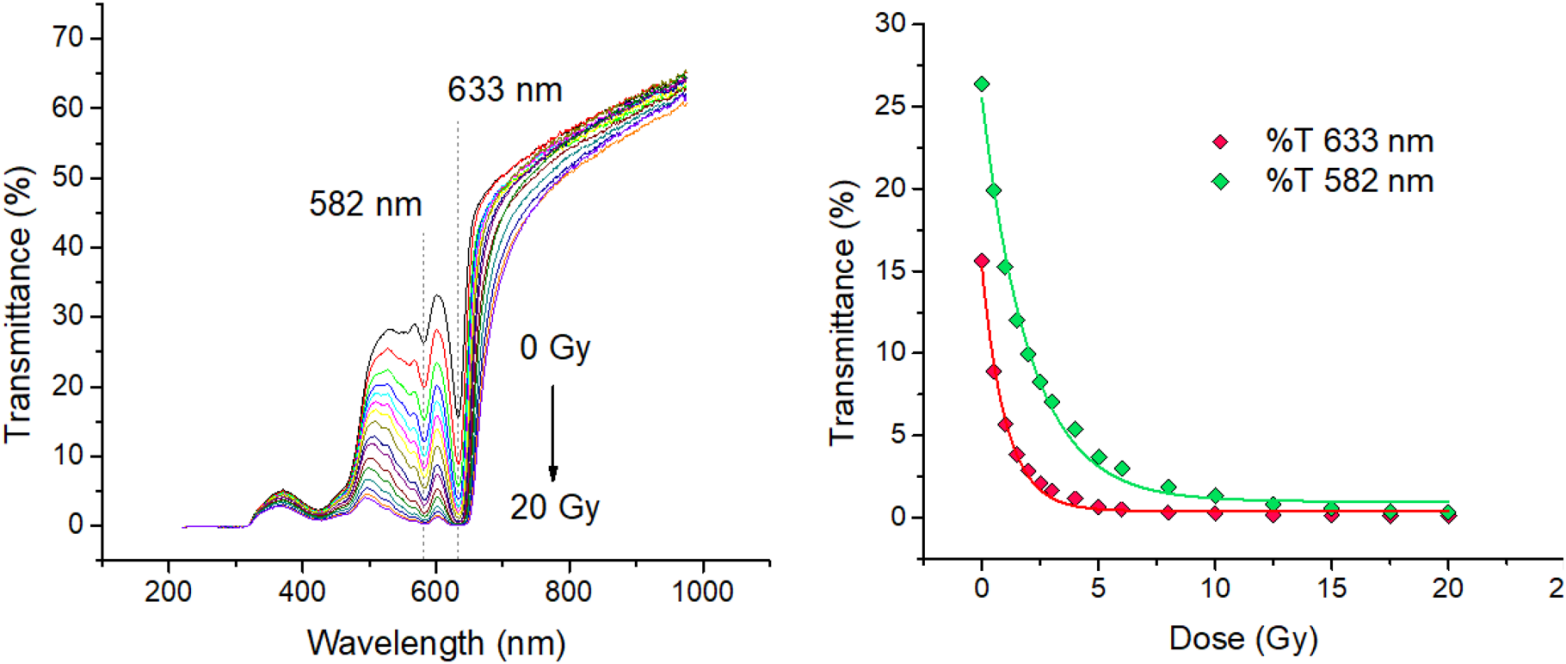
Left: Transmittance spectra of gafchromic EBT3 films irradiated with gammas at different doses. Right: Transmittance values evolution at 582 and 633 nm wavelength depending on the irradiation dose.

A Qe65 Pro Spectrometer controlled by OceanView software (OCEAN OPTICS) was used to delimit the transmittance spectra range of the gafchromic EBT3 films. The used light source was a deuterium‒halogen (DH‒2000‒BAL, Mikropack) lamp. The films were located between two optic fibers respectively connected to the light source and spectrometer. Spectroscopic analysis allowed the determination of the wavelength range for the measurement and thus to define the LED channels more appropriate for the measurement according to the percentage of light transmittance through samples. Figure 3, left, shows the transmittance spectra measured for a set of gafchromic EBT3 films. They were irradiated from 0 to 20 Gy with photons in a clinical gamma linear accelerator. The transmittance is particularly discernible in the red (633 nm) and green (582 nm) wavelength range. Figure 3, right, shows a considerable differentiation of the light transmittance as a function of the radiation dose below 10 Gy. Therefore, for the sensing purposes explained above, the red and green LED channels will be used as sensing signals in the tests performed hereinafter.

### Gafchromic EBT films

Absolute dose evaluations of the irradiated films were obtained following well stablished protocols for gafchromic EBT3 films^36,37^. The response of irradiated films was evaluated in terms of their OD changes before and after irradiation. To quantify it, we used a flat‒bed color scanner in transmission mode. These scanners measure the red, green, and blue colors of light transmitted by the films. It uses broad band fluoroscopic visible light sources. OD changes were assessed as a logarithm of the inverse transmission ^1^:

1 $${\text{netOD}} = {\text{OD}}_{{{\text{after}}}} - {\text{OD}}_{{{\text{before}}}} = - {\text{log}}_{10} \left( {{\text{I}}/{\text{Io}}} \right)$$

where Io and I represent the intensity of the light passing through the unexposed (control) and irradiated films respectively. Previous calibration curve to convert changes of OD into absolute dose is mandatory. It was performed by correlating the dose deposited inside the gafchromic EBT3 films with a reference dosimetry system. In particular, we used an ionization chamber in the CNA cyclotron, which was calibrated before under reference conditions^38^.

The useful range of gafchromic EBT3 films is up to 8 Gy if only the red absorption spectrum is used, but expanding to 100 Gy when using the three RGB channels. Minimum dose of 0.2 Gy was reported previously^31^. We irradiated a batch of films from 0.25 to 10 Gy with both red and green channels for the reasons detailed above.

Films handling was performed taking into account the recommendations provided by Task Group 55 of the AAPM^36^. A flat-bed red–green–blue (RGB) scanner (EPSON PERFECTION V700 photo scanner) was used for the readout at 75 dpi resolution. Images were saved in tagged image file format (tif) and later analysed with ImageJ. We followed the methodology described by Devic et al.^37^ and used the red and green channel, being the red one the one that showed the highest dose sensitivity.

It is worth noting that each films batch and scanner respond slightly different and therefore sensitivity curves might be different. Hence, they must be calibrated with a reference dosimeter and source for each film batch. Gafchromic EBT3 films were calibrated in a configuration where they were placed perpendicularly to the beam direction. Similar region of interest (ROI) were defined over the irradiated sample around the beam axis and mean pixel values of the transmission scan were recorded. NetOD was calculated as Eq. (1) with these values. Reading evaluations were calculated using an in‒house code regarding the calibration curves. Dose measurement uncertainties were below 3%.

### Proton beam line and irradiation configuration

A cyclotron facility (Cyclone 18/9 model) is installed at the National Centre of Accelerators (CNA, Seville, Spain). It has an external beam line for interdisciplinary research purposes and accelerates protons and deuterons at 18 and 9 meV, respectively. Particle beam proceeds horizontally to the experimental room via the beam transport system, consisting of a variable graphite slit, an XY magnetic steerer, a quadrupole doublet and a quadrupole singlet. A two meter thick concrete wall, through which a stainless steel vacuum pipe transports the beam, separates the cyclotron bunker from the experimental room, where the beam is finally extracted in air. Details can be found in Baratto‒Roldan A. et al.^38^.

Dosimetry evaluations were measured directly after the exit window with gafchromic EBT3 films. These films were positioned parallel to the surface of the ionization chamber and perpendicular to the incident proton beam. Rectangular pieces of films, cut from the same sheet and positioned with the same orientation with respect to the beam direction, were attached to the ionization chamber.

The beam is ensured to be homogeneous in the whole surface of the sample^38^. Maximum deviations from mean optical density were of the order of 2‒6% at the sample position over an area of 35 mm diameter.

Due to the energy lost in the exit window and into the air, the proton energy impinging on the gafchromic EBT3 films was lower than the nominal maximum (18 ± 0.14 MeV), namely 12.8 MeV with a standard deviation of 1.5% from the mean value and Gaussian distribution. Right after irradiation, we handled the irradiated films within the cyclotron control‒room.

### Calibration curve of Gafchromic EBT3 films

**Figure 4:**
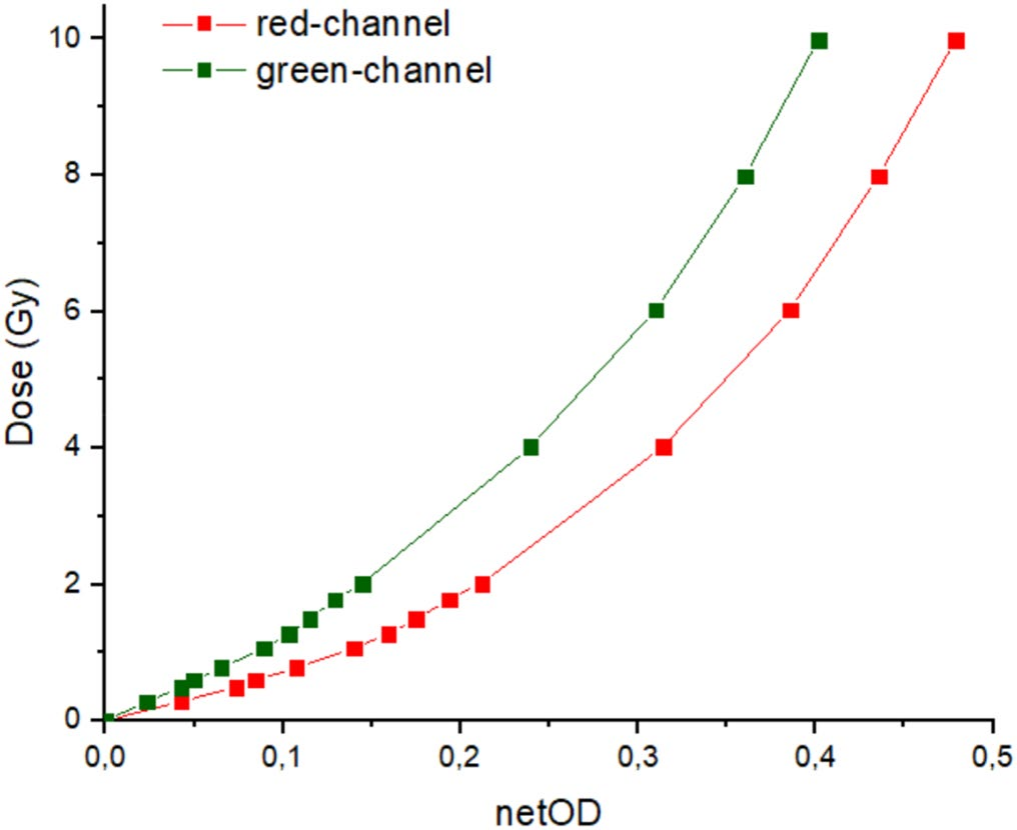
EBT3 calibration curves for one incident proton energy (12.8 MeV) for both red and green channels as films are scanned 48 h after irradiation.

The dose deposited in the film was evaluated through the proton fluence *f* measured with the ionization chamber, by using the calibration methodology detailed in ^38^. Figure 4 shows the calibration curve in the range between 0 and 10 Gy for a 12.8 MeV incident proton energy. It shows the characteristic non‒linear relation between the OD and the delivered dose.

## Results

A set of these EBT3 films were irradiated consecutively in the CNA with a 18 MeV proton beamline. Table 1 shows the delivered doses ranged from 0.25 to 10 Gy.

**Table 1:** Dose values of the irradiated films.

# film	Dose (Gy)
1	0.28 ± 0.01
2	0.49 ± 0.01
3	0.59 ± 0.01
4	0.76 ± 0.01
5	1.05 ± 0.01
6	1.26 ± 0.01
7	1.46 ± 0.01
8	1.75 ± 0.01
9	1.98 ± 0.01
10	3.96 ± 0.02
11	5.93 ± 0.03
12	7.86 ± 0.03
13	9.84 ± 0.04

Measurements were performed with two different methods: first, with the new approach based on the optical sensing system, films were put individually within the MOEMS to assess the voltage generated by it. These measurements were carried out in the cyclotron control room since gafchromic films are relatively insensitive to visible light. Afterwards, films were kept in the dark for better conservation. Measurements were also repeated at different times (1, 6, 24 and 48 h after irradiation) to evaluate possible changes over time. Secondly, with the standard method, films were put in a conventional optical densitometer at 24 and 48 hours after irradiation to evaluate the OD changes in the irradiated films.

Delivered doses were controlled by interlacing an ionization chamber in the proton beam path. It quantifies the charge, which is directly proportional to the dose as described in equation (2).

2 $${\text{D}}\,\left( {{\text{Gy}}} \right) = 0.0026 \cdot {\text{Q}}\,\left( {{\text{nC}}} \right)$$

Table 1 summarizes the delivered doses in each gafchromic EBT3 film. Additionally, two extra films were non-irradiated (control ones). Background ionization charge was always below 0.7 nC.

**Figure 5:**
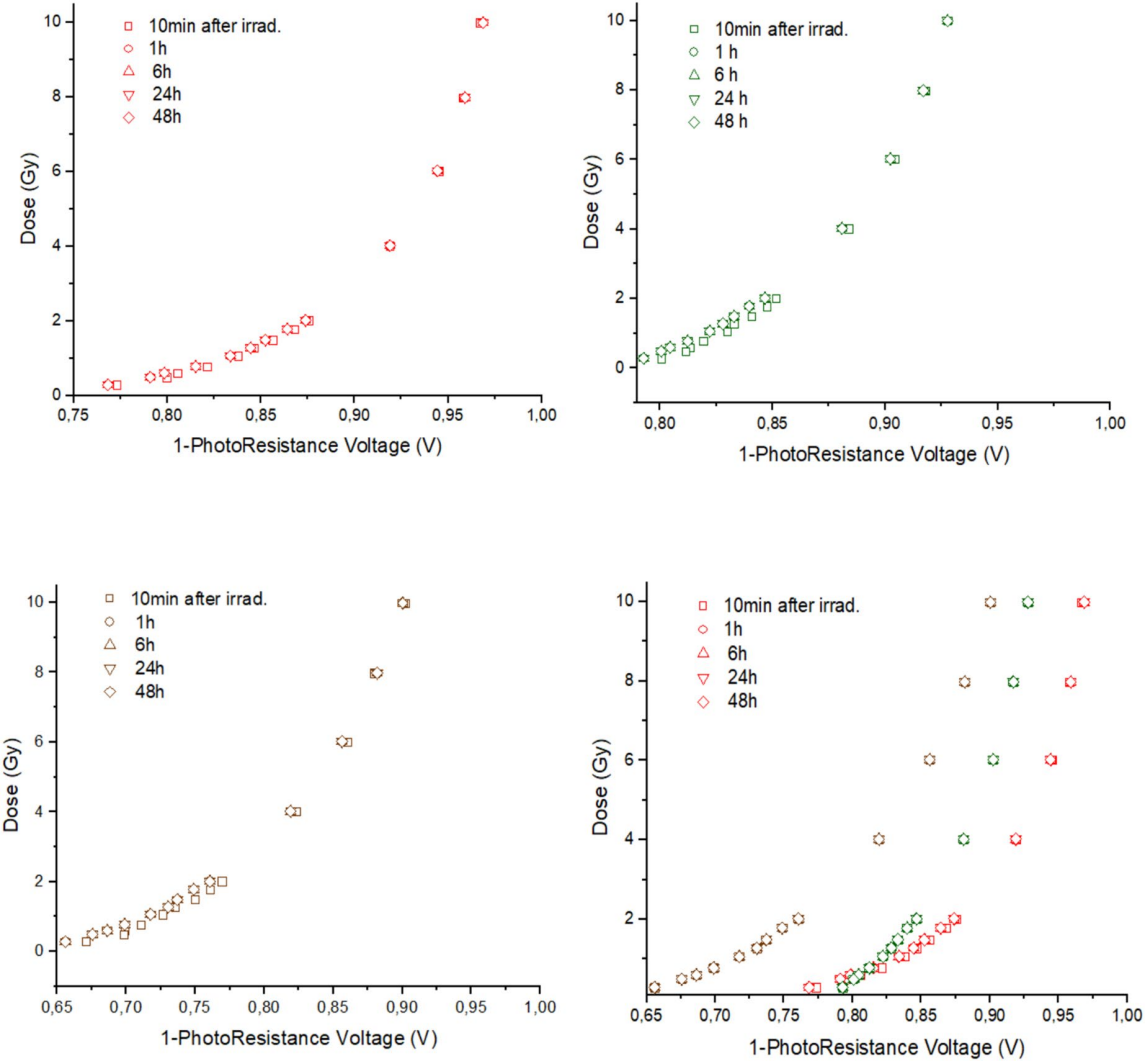
Photo-resistive voltage obtained for each light over time from all the irradiated samples with delivered doses from 0.25 to 10 Gy. From top-left to bottom-right: red, green, both red and green, and all channels respectively.

Figure 5 shows the dose obtained as a function of the photo‒resistive voltage generated in the MOEMS by the light transmitted through the sample. Similar tendencies as those obtained in Fig. 4 are displayed. They show a global non-linear behavior, although with two quasi‒linear regions corresponding at low (< 2 Gy) and high doses (> 2 Gy). Surprisingly, there were not significant differences in the photo‒resistive voltage values over time with the MOEMS. Even after 8 h post‒irradiation, which is the time assumed for stabilizing film darkening, there were not changes when both red and green LEDs, either independently or both together, were used. It means that the MOEMS setup may deliver a stable characterization parameter over time.

**Figure 6:**
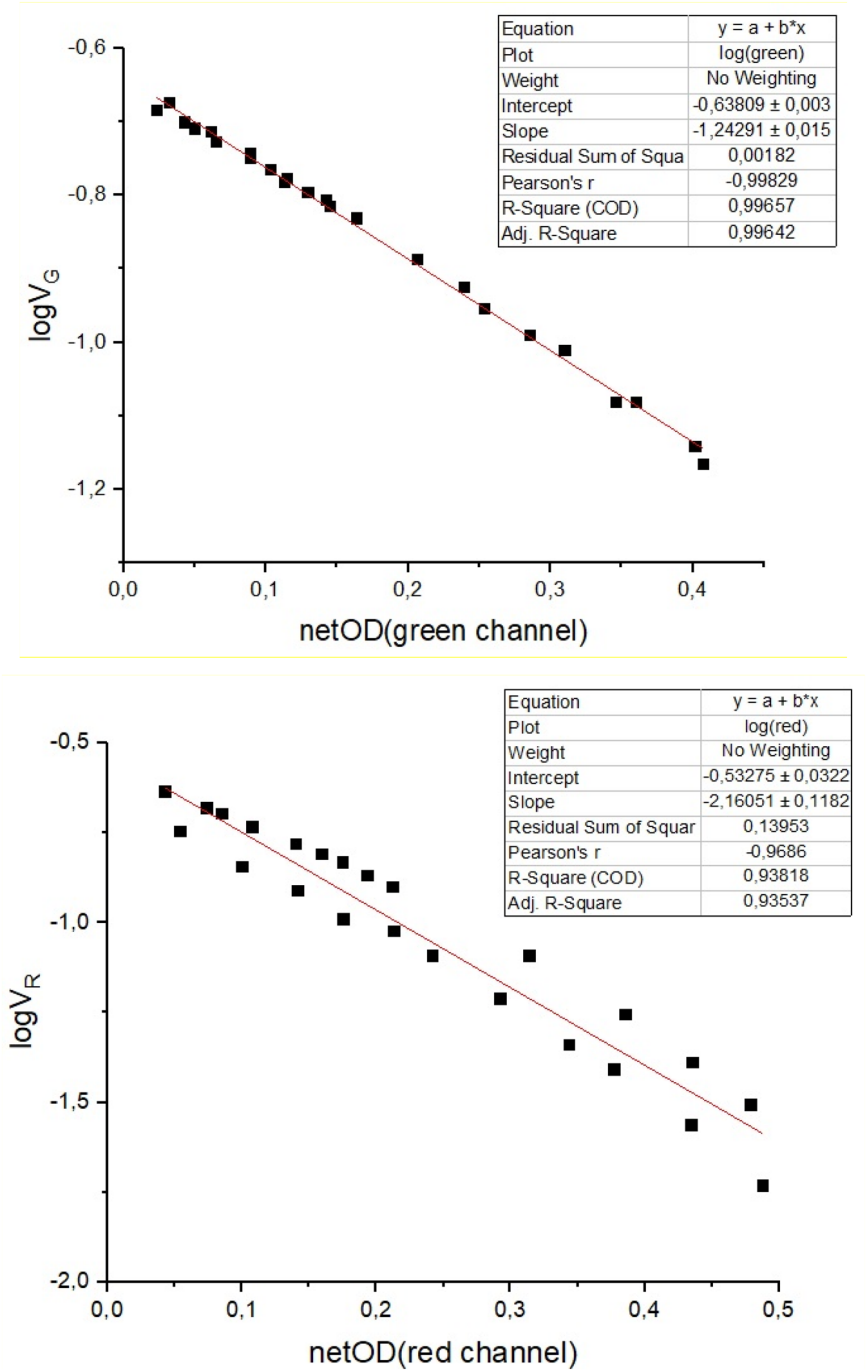
Photo-resistive voltage versus netOD obtained with both standard method for the green (top) and red (bottom) channels and using the MOEMS system for two independent gafchromic EBT3 film batches, namely those irradiated in the proton cyclotron and the photon LINAC respectively.

Additionally, an extra batch from another manufactured gafchromic EBT3 set was irradiated in a hospital by using gamma rays of a clinical LINAC from 50 to 1000 MU, i.e. 0.5 to 10 Gy. This set was also characterized with the two methods described in this work, i.e. with the scanning standard and the MOEM system respectively. The weak energy dependence of the gafchromic EBT3 films ensures the comparison of both sets irradiated with different radiation types. Figure 6 shows all photo-resistive voltage (V_R_) magnitudes versus the netODs for both channels and the two independent gafchromic EBT3 film batches. Figure 6, top, displays the values obtained for the green channel, while Fig. 6, bottom, shows those acquired for the red channel. Both independent sets followed a similar linear correlation between netOD and V_R_. We hypothesize the following rationale: according to the design of the MOEMS (see Fig. 1), the photo-resistive voltage registered is given by the following expression

3 $$V_{R} = V_{0} \times \frac{{R_{1} }}{{R_{1} + R_{2} }}$$

where V_0_ = 5 V is the voltage source, R_1_ = 1 kΩ is a fixed resistor, and R_2_ the photoresistor resistance. Regarding the *boundary* conditions, i.e. R_2_(dark) is 500 kΩ and R_2_(light saturation) is 500 Ω, R_2_ can be expressed as:

4 $$R_{2} \left( {k{\Omega }} \right) = \left( {\frac{1}{{\alpha I + \frac{1}{500}}} + 0.5} \right)$$

where I is the intensity of the light crossing the gafchromic EBT3 film and α is a constant. It can be related to the absorbance of the film through the Beer‒Lambert’s law of photometry, which states the relation between the attenuation of a monochromatic light and the properties of the material that the light crosses as:

5 $${\text{I}} = {\text{I}}_{0} \cdot 10^{{ - {\text{A}}}}$$

Being A the absorbance, which is the optical density netOD indeed (see Eq. (1)). It is valid up to an optical density of 2, which is within outcome range herein. In saturation conditions, i.e. the netOD tends to 0 and similarly I does to I_0_, the αI_0_ parameter can be evaluated starting from the V_R_ values of non-irradiated films. For example, we obtained V_R_ = 0.23 V and αI_0_ = 0.047 kΩ^-1^ for those values collected with the green channel for non-irradiated films. Therefore, the general equation of R_2_ as a function of the absorbance, A, is:

6 $$R_{2} \left( {k{\Omega }} \right) = \left( {\frac{1}{{0.04710^{ - A} + \frac{1}{500}}} + 0.5} \right) \approx \frac{1}{{0.04710^{ - A} }}$$

Except in saturation conditions, R_2_ >  > R_1_, and, consequently, we may deduce that the voltage read (see Eq. (3)) of the MOEMS is:

7 $$V_{R} \approx V_{0} \times \frac{{R_{1} }}{{R_{2} }} = V_{0} \times 0.047*10^{ - A} = 0.235*10^{ - A}$$

It is worth realizing that by using the conventional method, i.e. a flat scanner, light impinges perpendicular on the film surface. In contrast, with the MOEMS setup, due to the more random fitting of the gafchromic EBT3 film within the sample cavity, light may cross that film with a certain angle. It might increase the light optical path and therefore increasing the absorbance. Thus, for an initial I_0_, the MOEMS and flat scanner systems could measure a different value of the absorbance depending on the light wavelength used. In the case of using MOEMS setup, the *A* value (A_MOEMS_) has to be considered as the attenuation coefficient multiplied by the optical path, i.e.:

8 $$A = \int_{0}^{l} a\left( z \right)dz$$

where a(z) is the decadic attenuation coefficient and *l* the thickness of the film that the light crosses. If the a(z) is uniform along the path, Eq. (8) evolves into: A = a∙ l. In turn, it is correlated with that obtained with the flat scanner (A_FS_) by a constant as: A_MOEMS_ = β · A_FS_, being β > 1. Hence, considering logarithms in the Eq. (7), we obtain a relationship between the V_R_ and the absorbance (netOD) measured for the green channel as:

9 $${\text{log}}\left( {{\text{V}}_{{\text{R}}} } \right)\left| {_{{{\text{green}}}} = - 0.63 - \beta_{{{\text{green}}}} \cdot {\text{A}}_{{{\text{FS}}}} = - 0.63 - \beta_{{{\text{green}}}} \cdot {\text{netOD}}} \right|_{{{\text{green}}}}$$

The first term of Eq. (9) is in very good agreement with the intercept slope of the linear fitting for the measurements performed with the green channel (see Fig. 6, top).

Same reasoning than above for the red channel (V_R_ = 0.264 V and αI_0_ = 0.055 kΩ^−1^ for those values collected for non-irradiated films) yields:

10 $${\text{log}}\left( {{\text{V}}_{{\text{R}}} } \right)\left| {_{{{\text{red}}}} = - 0.56 - \beta_{{{\text{red}}}} \cdot {\text{A}}_{{{\text{FS}}}} = - 0.56 - \beta_{{{\text{red}}}}\cdot {\text{netOD}}} \right|_{{{\text{red}}}}$$

Good agreement within the error tolerances was also found with the intercept slope of the linear fitting for the measurements performed with the red channel (Fig. 6, bottom). The experimental values of β for the green and red channels are 1.243 ± 0.015 (R^2^ = 0.996) and 2.16 ± 0.11 (R^2^ = 0.93) respectively, according to the linear fit displayed in Fig. 6. Considering that the green and red photons in the MOEMS traverse the same optical path, the larger β value obtained for the red channel can be ascribed to the lower transmittance of the films to the 633 nm wavelength in comparison to the 582 nm (see Fig. 3).

To determine the dose uncertainty of MOEM system, the optical density of the films was measured using a conventional scanner at 10 minutes and 48 hours after irradiation. With the help of the calibration curve, we evaluate that the difference in OD corresponds to an uncertainty in dose of 7% at 2 Gy and it decreases to 5% at 10 Gy.

Certainly, the linear correlation between V_R_ and netOD data for independent gafchromic EBT3 batches was stronger for the values obtained with the green channel. It means that using only that channel and after obtaining the calibration curve, the dose delivered can be obtained in situ and in real-time with the MOEM system, but with a dose uncertainty up to 7%.

## Discussion

Nowadays, one of the main drawbacks of gafchromic films as clinical dosimeters is the post-irradiation time delay for evaluating optical density changes due to radiation. From the radio physicist standpoint, regarding fast QA requirements daily, it might be more useful to use instantaneous readings even to the detriment of dose uncertainty increasing. In this framework, the feasibility of our Micro-Opto-Electro-Mechanical system (MOEMS) for accurate dosimetry has been investigated. We found that the MOEMS photo-resistive voltage works as a feasible physical parameter that allows us to quantify directly dose and avoid data post-processing and time-delay.

As it is shown in Figure 5, there is a negligible change in the photo-resistive voltage values for any of the used LED channels over time. It means that these values should be considered constant over the time and, therefore, the initial magnitude (just after irradiation) may be used to determine the irradiation dose. Thus, *in situ* measurements can be correlated with the delivered dose without time-delay when using the MOEMS setup, if the calibration curve is obtained previously. However, we notice that, as the optical density of the gafchromic EBT3 films changes slightly over time after irradiation, the insensitivity of the MOEMS response to this variation implies a limitation of this methodology to discern nearby doses.

Likewise, we have found a direct relationship between the photo‒resistive voltage of the MOEMS and the netOD obtained with the standard flat scanning method for the two light channels used (see Eqs. (9) and (10)). These relations do not depend on either the gafchromic EBT3 film batch or the particle type as we demonstrated by comparing two independent film batches with gamma and proton irradiation respectively, as it is detailed above. It means that, once the calibration curve of a particular set of gafchromic EBT3 films is acquired, delivered dose can be directly determined by measuring with the green channel of the MOEMS, which showed direct correlation with the corresponding netOD (Fig. 6, top). This configuration allows a considerable time saving and the in situ verification of the delivered dose in the patients or phantoms used in clinical scenarios.

Some improvements of the MOEMS are necessary although. For example, (i) it is indispensable a mechanical adjustment in the sample cavity to avoid absorbance changes due to the positioning tilt; (ii) a new design of the MOEMS modules that would allow radiation to impinge directly in gafchromic films integrated inside is preferable; (iii) there is still room for increasing the photo-resistance sensitivity and thus reducing the dose uncertainty; (iv) a minimal invasive design is preferable for potential on-line dose continuous monitoring. These developments are object of ongoing studies. For example, considering the patient irradiation conditions in real‒time, we are currently working in several improvements^30^. Namely, based on the presented work herein, we are working in (i) an ultrathin optical multiplex sensor to be deposited over the patient skin as a wearable system. It will contain tens of micro photodetectors customized. Several spread light emitting diodes will be integrated in an attached flexible printed circuit and placed laterally to avoid interposing between the radiation and the radiochromic film. The whole sensitive area will cover 10 cm^2^. It will allow us to characterize heterogeneous dose distributions in the patient skin in situ and real‒time and high dose gradients measured; (ii) a readout integrated circuit that will comprise micro‒antennas in communication to correlate the associated voltage changes with doses (using the previously calibration curve values under reference conditions with a radiation beam quality and following an established reference dosimetry protocol, for example TRS 398^39^); and (iii) obtaining the online data acquisition as well as the corresponding post‒processing of all the outcomes with an in‒house Graphical User Interface with an open‒source code, e.g. Python. It will transmit by wireless communication the results to the control room of treatments.

Consequently, even if the optical density has been historically a quantity of choice to dosimetry characterization gafchromic EBT3 films, the V_R_ opto‒electro parameter delivered by the MOEMS can be used for simpler and faster processing to evaluate the deposited dose in a wide clinical applications range (with delivered doses lower 10 Gy). However, the dose uncertainty with the current MOEM system is higher (≤ 7%) than with the standard method (≤ 3‒7%)^40^. Even so, enhancements for reducing dose uncertainties are required by increasing the sensitivity of our photodetectors.

For the best of our knowledge, the only other alternatives in real-time are based on optical fibers^32–35^, where the films are located on the tip of fibers, which might not pursue the guidelines for radiochromic films handling^36^. In these systems the light is backscattered after passing into the films and the sent to a spectrometer. For example, the Casolaro’s setup consists in both large and heavy light spectrometer and power light source that require a warm‒up time and it has a single‒spot resolution. These features make this setup difficult for daily dose monitoring, specially whether some spatial resolution is needed. In contrast, thanks to the high electronic versatility of the MOEM system, it would allow for not only using standard interchangeable radiochromic film pieces, but also having a portable setup whose second module (light‒dependent resistor components) can be extended to reach multiple sensitive spots, i.e. high spatial resolution. Table 2 summarizes the main features between the few systems available based on optic fibers, the current MOEMs presented herein, and the advanced prototype in which we are working in with the improvements listed above.

**Table 2:** Comparison of the main features of current active dosimeters based on gafchromic films.

Method	Resolution	Equipment	Cost	Multiplex	Flexible	Real time
Standard	High	Flat scanner	Low	Yes	No	No
Fiber optics	Poor	Light spectrometer power light source	High	No	No	No
MOEMs	Medium	Arduino	Low	Yes	No	Yes
Flexible MOEMs	High	Tailored MOEMs & readout	Medium	Yes	Yes	Yes

Finally, radiochromic films are widely used in other fields, e.g. beam diagnostics, radiation-induced sterilization for food and medical devices, electronics radiation damage. The device and methodology presented herein can be too employed in those fields.

## Conclusions

We present the first direct measurements of dose in gafchromic EBT3 films with a micro-opto-electro-mechanical system in particle beams. The feasibility of accurate dosimetry using a portable optical analysis system in radiotherapy was evaluated. This system offers a very fast solution for dose measurement and verification in radiotherapy. We point out the use of this system for in situ determination of delivered dose in radiotherapy environments with considerable time reduction.

Likewise, this system accomplishes the guidelines for radiochromic films handling and analysis. It means that it is possible to use the integrated compact MOEM system in situ without post-irradiation delay provided the calibration curve is available, which is the case in all the QA clinical facilities. That is, our system allows us to take advantage of the current gafchromic films and overcome traditional limitations. Currently we are performing a portable multi‒MOEMs system to increase the spatial resolution.

To the best of our knowledge, there is not previous works regarding this new approach based on MOEMS and this is the first trial to overcome the time‒delay in radiochromic film analysis by turning a passive detector into a potential active sensing system.

## Data Availability

The data analysed during the current study are available from the corresponding author.
